# Measurement properties, interpretability and feasibility of instruments measuring oral health and orofacial pain in dependent adults: a systematic review

**DOI:** 10.1186/s12903-022-02235-w

**Published:** 2022-05-25

**Authors:** Fahad A. BaHammam, Giles I. McCracken, Rebecca Wassall, Justin Durham, Bana Abdulmohsen

**Affiliations:** 1 School of Dental Sciences, Faculty of Medical Sciences, Newcastle University, Newcastle upon Tyne, UK; 2 College of Dentistry, King Saud bin Abdulaziz University for Health Sciences, Riyadh, Saudi Arabia

**Keywords:** Oral health, Orofacial pain, Dependent adults, Measurement properties, Interpretability, Feasibility

## Abstract

**Background:**

Dependent adults have been shown to have a greater experience of oral health deterioration and orofacial pain. This is partly because their non-dental caregivers may not easily identify oral health problems and orofacial pain experienced by them. Thus, this systematic review aimed to investigate measurement properties, interpretability and feasibility of instruments assessing oral health and orofacial pain in dependent adults, which can be used by the non-dental caregivers to establish oral care plans for those who are dependent upon them.

**Methods:**

Seven bibliographic databases were searched: MEDLINE, Embase, CINAHL, CENTRAL, HTA, OATD and OpenGrey. Citations and reference lists of the included studies were also manually searched. Two authors independently screened titles and abstracts, and then full texts. A quality assessment of included studies was conducted independently by two authors using the COSMIN Risk of Bias checklist. The best evidence synthesis method was used to synthesise results from different studies for each measurement property per measurement instrument by integrating the overall rating for each measurement property per measurement instrument with its quality level of evidence.

**Results:**

Nineteen eligible studies were included, which reported the development, measurement properties’ evaluation, interpretability and feasibility of nine oral health and three orofacial pain measurement instruments. Methodological quality of the included studies ranged from very good to inadequate. None of the identified measurement instruments has been adequately and comprehensively tested.

**Conclusions:**

While several measurement instruments were identified in this systematic review, more evidence is needed to be able to more comprehensively evaluate these instruments. Among those identified, the OPS-NVI demonstrated sufficient construct validity, while the OHAT and the THROAT demonstrated sufficient reliability. These instruments therefore have potential for future use with more confidence once other measurement properties, interpretability and feasibility have been sufficiently tested and evaluated.

**Supplementary Information:**

The online version contains supplementary material available at 10.1186/s12903-022-02235-w.

## Background

Dependency on care is a social construct that does not represent a personal attribute of individuals, but a social relationship between them [1]. There are two key characteristics of any care dependency relationship: a dependent adult lacking power in the relationship especially in making decisions; and a lack of the capacity to pay back [2, 3]. Care dependency can promptly occur because of physical- or mental-related conditions causing disability [4]. However, it can also develop gradually in older adults due to frailty or comorbidity [5]. The relevance of oral health to dependent adults could be inferred from the general impact of dependency on them. One of the major dependency consequences is the deterioration in the self-care domain, which include oral care [6]. In addition, dependency can cause a decline in the economic status of the dependent adults and their families, which may limit their access to optimal dental services [7].

In light of the above, it might be unsurprising that dependent adults who are reliant on others for self-care have been reported to experience many oral health problems, which include deterioration in their dentition, oral soft tissues and dentures [8–10]. In addition, several studies have reported that orofacial pain is a common problem among them, with approximately 1 in 4 dependent adults being affected by it [10–12].

Oral health problems and orofacial pain experienced by dependent adults could partially be explained by the challenging nature of providing them with daily oral care, which is particularly the case if caregivers are unable to easily identify oral health problems and orofacial pain of those who are dependent upon them [13, 14]. This is supported by several reports from caregivers who have voiced their need for an oral health measurement instrument that could help them establishing oral care plans for dependent adults [15–17]. It is perhaps, therefore, no surprise that a number of measurement instruments have been specifically developed for this purpose [18]. The Brief Oral Health Status Examination (BOHSE) Index is an example of such an instrument that was developed to be used in care homes [19]. It was modified 10 years later to improve its feasibility and usability [20]. Another measurement instrument is The Holistic and Reliable Oral Assessment Tool (THROAT) that was developed to be used in stroke wards [21].

Evidence about these instruments’ performance, however, is dispersed in the scientific literature, and therefore, accessing this evidence is not easy. This can negatively affect the caregivers' ability to reach a sound and scientific judgment about the use of these instruments, which could partially explain why they have not been widely used in clinical settings [22]. A systematic review about these measurement instruments was therefore designed to identify the published evidence about these measurement instruments and establish an evidence-based decision about the best available instrument for use.

## Methods

### Aim

To systematically identify measurement instruments assessing oral health or orofacial pain in dependent adults that have been validated to at least some extent and evaluate these instruments’ measurement properties, interpretability and feasibility.

### Protocol and reporting

The protocol of this systematic review was registered in the International Prospective Register of Systematic Reviews (PROSPERO) database CRD42017073404 [23]. This systematic review is reported in accordance with the Preferred Reporting Items for Systematic Reviews and Meta-Analyses (PRISMA) statement [24].

### Literature search strategy

Seven electronic databases were searched from inception to the 9th of October 2017 and were later updated on the 1st of August 2019 and 25th of February 2022: MEDLINE, CINAHL, Embase, Cochrane Central Register of Controlled Trials (CENTRAL), Health Technology Assessment (HTA), Open Access Theses and Dissertations (OATD) and OpenGrey. The search strategy was first developed for the MEDLINE database using relevant keywords and Medical Subject Headings (MeSH) terms (Additional file 1). Then, it was translated and revised appropriately for the other databases considering the differences in thesaurus terms and syntax rules. The search strategies have focused on three key elements: the constructs of oral health and orofacial pain, dependent adults and measurement properties. The “measurement properties” component of the search strategy was adopted from a previously developed methodological search filter, which has been demonstrated to be highly sensitive and able to retrieve more than 97% of studies related to measurement properties [25]. The electronic databases searches were restricted to English language.

Other search methods have been utilised including a bibliographic manual hand search for the included studies. In addition, citation searches for the included studies were carried out using Scopus and Web of Science citation indices. Finally, Scopus and Web of Science were searched using the name of the identified measurement instruments and their abbreviations from the previously described search methods to identify further studies for inclusion on the 8^th^ of August 2019 and 4^th^ of March 2022.

### Selection and eligibility criteria

Two authors (FB and BA) independently screened the titles and abstracts and then full texts to select eligible studies based on predetermined inclusion and exclusion criteria. Disagreements were resolved through discussion between the two authors (FB and BA) and when necessary, with the third author (GM). The inclusion and exclusion criteria for selecting eligible studies in this systematic review followed the PICOS criteria:

- Participants: Eighteen years or older who need or receive support/assistance due to a reduction in mental capacity or physical capability
- Interventions: Measuring oral health or orofacial pain
  The concept of oral health in this review was defined according to the Strategy Group in the National Health Service definition [26], which stat that oral health is *“a standard of health of the oral and related tissues that enables an individual to eat, speak, and socialise without active disease, discomfort, or embarrassment, and that contributes to general wellbeing”*. On the other hand, orofacial pain has been defined according to International Classification of Orofacial Pain, which classified it into orofacial pain attributed to disorders of dentoalveolar and anatomically related structures, myofascial orofacial pain, temporomandibular joint pain, orofacial pain attributed to lesion or disease of the cranial nerves, orofacial pains resembling presentations of primary headaches and idiopathic orofacial pain [27].
- Comparators: This component was not considered because it is not applicable for systematic reviews evaluating measurement properties of measurement instruments
- Outcomes: The primary outcomes in this systematic review are three measurement properties (validity, reliability, and responsiveness), which are further divided into nine measurement properties, while interpretability and feasibility are the secondary outcomes
- Studies: Study published in the English language that are available in full text, which either describe the development of an original instrument to assess oral health or orofacial pain in dependent adults or evaluate measurement properties, interpretability or feasibility of such an instrument

### Quality assessment

Quality assessment of the included studies’ methods was undertaken independently by two authors (FB and BA) using the COSMIN Risk of Bias checklist [28]. The COSMIN Risk of Bias checklist consists of 10 boxes, in which each box evaluates the quality of the method used to assess a specific measurement property of that instrument. Each box is evaluated by assessing 3 to 35 items and the evaluation’s score for each item could be one of four scores: very good, adequate, doubtful or inadequate. The “worst score counts” method was used when evaluating each box so that the overall score for a particular box was determined by the lowest rating of any item in that box. In the case of any disagreement, the decision was made through discussion between the two authors (FB and BA) and with the third author (GM) where necessary.

### Data extraction

Data extraction was done independently by two authors (FB and BA) using a predesigned form in the Microsoft Excel ® software. Any disagreement was resolved through discussion between the two authors (FB and BA) and with the third author (GM) where necessary. Information extracted from the included studies was about: participants' characteristics, instruments' characteristics and results on instruments’ measurement properties, interpretability and feasibility.

### Data synthesis

Data synthesis in this systematic review was undertaken through several steps. First, the results on each measurement property from each study were rated as either sufficient, insufficient or indeterminate, using predetermined criteria that are presented in Additional file 2. It must be noted that any included study could have evaluated a certain measurement property several times in the same population or in more than one population. Several evaluations that were undertaken in multiple populations were considered as different studies and each evaluation was rated separately. On the other hand, multiple evaluations that were undertaken on the same population were considered as a single study and therefore all different evaluations were collectively rated at once. To resolve any inconsistency in the results in the last scenario, a sufficient or insufficient rating was assigned if 75% or more of the results were in accordance with sufficient or insufficient criteria. Otherwise, an inconsistent rating was assigned.

The measurement properties’ ratings from the last step were summarised to come to an overall evaluation of each measurement property for each identified measurement instrument from all contributing studies. If ratings of an instrument’s measurement property from different studies were consistent, the same rating was assigned for the overall rating of the instrument’s measurement property. However, if ratings of an instrument’s measurement property from different studies were inconsistent, the overall rating of the instrument’s measurement property was based on the majority of consistent results (≥ 75%). If no majority of consistent results could be identified, ratings were not summarised, and an overall inconsistent rating was given.

The quality of evidence supporting the overall rating for each measurement property per measurement instrument was graded based on the Grading of Recommendation Assessment, Development and Evaluation (GRADE) approach [29, 30]. This grading process has four potential outcomes: high quality level; moderate quality level; low quality level; and very low quality level. The level of evidence for an overall rating was always graded as being of a high quality level and was only downgraded where there was a concern in one or more of the GRADE factors, which are risk of bias, inconsistency, imprecision and indirectness.

Last, best evidence synthesis was carried out by integrating the overall rating for each measurement property per measurement instrument with its quality level of evidence that was established utilising the GRADE approach.

## Results

### Results of literature searches

**Fig. 1:**
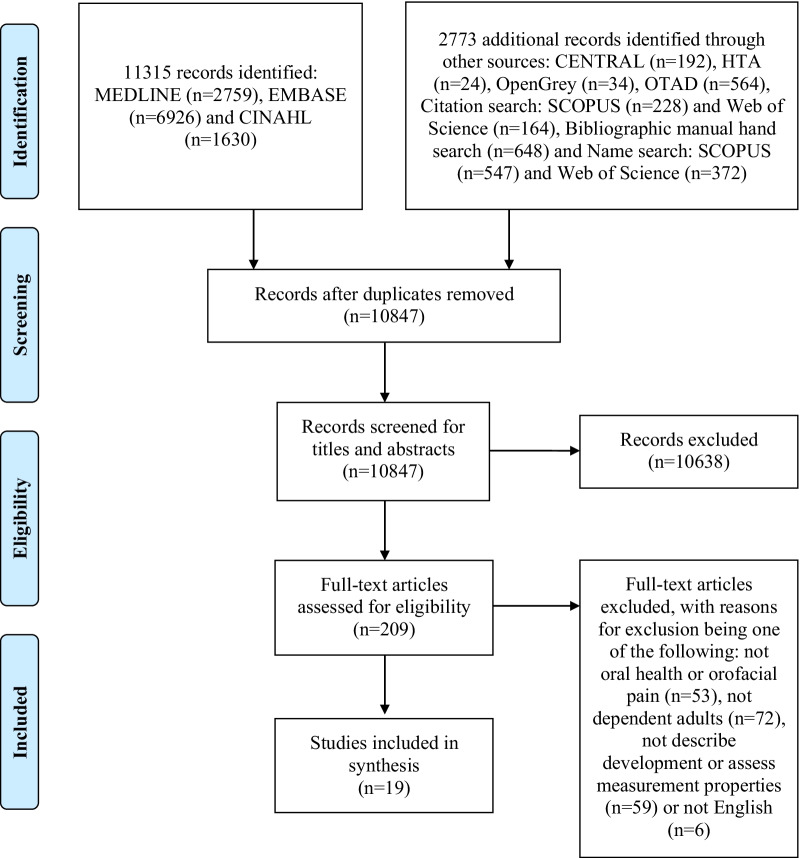
PRISMA flow diagram summarising the retrieval, screening and selection processes

The literature search retrieved 14,088 studies. After duplicates were removed, and titles and abstracts and then full texts were screened, 19 studies met the inclusion criteria and were therefore included. Figure 1 presents a PRISMA flow diagram that summarises the retrieval, screening and selection processes.

### Characteristics of included studies and measurement instruments

The 19 included studies can be divided into two groups. The first group consists of 14 studies that described the development and evaluation of measurement properties, interpretability and feasibility of nine oral health measurement instruments (Table 1). The second group consists of five studies that described the development and evaluation of measurement properties, interpretability and feasibility of three orofacial pain measurement instruments (Table 1). The table in Additional file 3 presents the main characteristics of the identified oral health and orofacial pain measurement instruments.

**Table 1:** Characteristics of the included studies

Instrument name	Author (Year), Country	Study aim	Dependency cause (Setting)	Sample Size
*Oral health measurement instruments*				
BOHSE	Kayser-Jones et al. (1995), USA [19]	Development and reliability assessment	Age-related (Care homes)	100
MPS	Henriksen (1999), Norway [43]	Reliability assessment	Age-related and mental-related (Care homes)	Study (A): 24 Study (B): 20
THROAT	Dickinson et al. (2001), UK [21]	Development and reliability assessment	Physical-related (Hospital)	50
	Mckenzie (2015), UK [50]	Construct validity assessment	Physical-related (Hospital)	32
ROAG	Andersson et al. (2002), Sweden [51]	Development and reliability assessment	Physical-related (Hospital)	133
	Konradsen et al. (2014), Denmark [44]	Reliability assessment	Physical-related (Hospital)	148
OHAT	Chalmers et al. (2005), Australia [20]	Development, construct validity and reliability assessment	Age-related (Care homes)	455
	Simpelaere et al. (2016), Belgium [52]	Reliability assessment	Age-related and physical-related (Care homes and hospital)	132
	Şahin et al. (2019), Turkey [53]	Reliability assessment	Age-related (Care homes)	100
	Klotz et al. (2020), Germany [37]	Reliability assessment	Age-related (Care homes)	18
OHI	Liétard et al. (2013), France [54]	Development	Age-related (Care homes)	Not applicable
OAS	Yanagisawa et al. (2017), Japan [36]	Development, and reliability assessment	Age-related (Care homes)	45
OHSTNP	Tsukada et al. (2017), Japan [39]	Development, and reliability assessment	Age-related (Care home)	57
BOE	Kothari et al. (2022), Denmark [55]	Construct validity assessment	Physical- and metal-related (Hospital)	90
*Orofacial pain measurement instruments*				
FACS	Hsu et al. (2007), USA [56]	Development, construct validity and responsiveness assessment	Dementia (Dental clinics)	10
MOBID	Toxopeus et al. (2017), Netherlands [47]	Reliability assessment	Dementia (Care home)	11
OPS-NVI	De Vries et al. (2016), Netherlands [48]	Reliability assessment	Dementia (Care homes)	153
	Delwel et al. (2018), Netherlands [49]	Construct validity and reliability assessment	Dementia (Care homes and hospital)	348
	van de Rijt et al. (2019), UK [57]	Construct validity assessment	Dementia (Hospitals)	56

*BOE* Bedside Oral Examination, *BOHSE* Brief Oral Health Status Examination, *MPS* Mucosal-Plaque Score, *THROAT* The Holistic and Reliable Oral Assessment Tool, *ROAG* Revised Oral Assessment Guide, *OHAT* Oral Health Assessment Tool, *OHI* Oral Health Index, *OAS* Oral Assessment Sheet, *OHSTNP* Oral Health Screening Tool for Nursing Personnel, *FACS* Facial Actions Coding System, *MOBID* Mobilization–Observation–Behaviour–Intensity–Dementia for mouth care, *OPS-NVI* Orofacial Pain Scale for Non-Verbal Individuals

### Findings about measurement properties

Table 2 presents the main findings about methodological quality, individual ratings and overall ratings of measurement properties. The methodological quality of the included studies ranged from very good to inadequate. Most of the oral health measurement instruments’ studies have shown a doubtful methodological quality and only one study has shown very good methodological quality. Adequate and doubtful evaluations were the most common evaluations among orofacial pain measurement instruments studies.

**Table 2:** Methodological quality and ratings of measurement properties of measurement instruments

		Construct validity			Reliability			Responsiveness		
Instrument name	Study	Method quality	Rating	Overall rating	Method quality	Rating	Overall rating	Method quality	Rating	Overall rating
*Oral health measurement instruments*										
BOHSE	[19]	–	–	–	Doubtful	Inconsistent	Inconsistent	–	–	–
MPS	[43]	–	–	–	Doubtful	Insufficient	Inconsistent	–	–	–
	[43]	–	–		Doubtful	Sufficient		–	–	
THROAT	[21]	–	–	Insufficient	Adequate	Sufficient	Sufficient	–	–	–
	[50]	Adequate	Insufficient		–	–		–	–	
ROAG	[51]	–	–	–	Doubtful	Insufficient	Insufficient	–	–	–
	[44]	–	–		Doubtful	Insufficient		–	–	
OHAT	[20]	Inadequate	Inconsistent	Inconsistent	Doubtful	Sufficient	Sufficient	–	–	–
	[52]	–	–		Very good	Sufficient		–	–	
	[53]	–	–		Doubtful	Sufficient		–	–	
	[37]	–	–		Doubtful	Sufficient		–	–	
OHI	[54]	–	–	–	–	–	–	–	–	–
OAS	[36]	–	–	–	Doubtful	Inconsistent	Inconsistent	–	–	–
OHSTNP	[39]	–	–	–	Doubtful	Sufficient	Sufficient	–	–	–
BOE	[55]	Inadequate	Insufficient	Insufficient	–	–	–	–	–	–
*Orofacial pain measurement instruments*										
FACS	[56]	Adequate	Insufficient	Insufficient	–	–	–	Doubtful	Sufficient	Sufficient
MOBID	[47]	–	–	–	Doubtful	Insufficient	Insufficient	–	–	–
OPS-NVI	[48]	–	–	Sufficient	Adequate	Inconsistent	Inconsistent	–	–	–
	[49]	Doubtful	‡Not rated		Inadequate	Sufficient		–	–	
	[57]	Adequate	Sufficient		–	–		–	–	

‡The construct validity of the OPS-NVI in this study was evaluated based on the sensitivity and specificity. This was not incorporated into this systematic review because neither the research team nor the review team has defined a hypothesis in advance about their sufficient criteria

None of the results about construct validity of oral health measurement instruments were rated as sufficient. Regarding ratings the reliability results of the oral health measurement instruments, three of the instruments (i.e. THROAT, Oral Health Assessment Tool (OHAT) and Oral Health Screening Tool for Nursing Personnel (OHSTNP)) have shown to have an overall sufficient reliability. The Orofacial Pain Scale for Non-Verbal Individuals (OPS-NVI) was the only measurement instrument that was rated as having sufficient construct validity among orofacial pain measurement instruments. The reliability for all orofacial pain measurement instruments were rated overall as insufficient or indeterminate. The Facial Actions Coding System (FACS) was the only measurement instrument that demonstrated sufficient performance in responsiveness overall rating.

### Best evidence synthesis

Best evidence synthesis for the findings about the oral health and orofacial pain measurement instruments is presented in Table 3. OHAT showed the best performance among oral health measurement instruments by demonstrating high evidence of sufficient reliability. OPS-NVI showed the best performance among orofacial pain measurement instruments by demonstrating moderate evidence of sufficient construct validity.

**Table 3:** Best evidence synthesis of measurement instruments

Instrument name	Construct validity	Reliability	Responsiveness
*Oral health measurement instruments*			
BOHSE	NAs	±	NAs
MPS	NAs	±	NAs
THROAT	?	+	NAs
ROAG	NAs	–	NAs
OHAT	±	+ + +	NAs
OHI	NAs	NAs	NAs
OAS	NAs	±	NAs
OHSTNP	NAs	?	NAs
BOE	?	NAs	NAs
*Orofacial pain measurement instruments*			
FACS	?	NAs	?
MOBID	NAs	?	NAs
OPS-NVI	+ +	±	NAs

+  +  + or – – –: high evidence of sufficient or insufficient results, +  + or – –: moderate evidence of sufficient or insufficient results, + or –: low evidence of sufficient or insufficient results, ?: unknown due to evidence with very low quality, ± : unknown due to inconsistent results, NAs: Not Assessed

### Interpretability and feasibility outcomes

The interpretability of the included measurement instruments was evaluated based on the distribution of the instruments’ scores, obtained when these instruments used to assess oral health or orofacial pain in dependent adults, in the form of mean and standard deviation. The presence and absences of floor and ceiling effects were also utilised to give more insight into the interpretability of the instruments. Findings about the interpretability are presented in Table 4.

**Table 4:** Findings about interpretability of measurement instruments

Instrument name	Study	Scores distribution Mean (standard deviation)	Floor and ceiling effect
*Oral health measurement instruments*			
BOHSE	[19]	4.29 (2.87) out of 20	–
MPS	[43]	4.48 out of 8	–
THROAT	[21]	–	–
	[50]	–	–
ROAG	[51]	10.16 (3.63) out of 24	–
	[44]	–	–
OHAT	[20]	2.54 out of 16	–
	[52]	–	–
	[53]	–	–
	[37]	6.70 (2.80) out of 16	–
OHI	[54]	1.40 out of 8	–
OAS	[36]	–	–
OHSTNP	[39]	–	–
BOE	[55]	11.2 (3.0) out of 24	–
*Orofacial pain measurement instruments*			
FACS	[56]	28.80 (9.60) †	–
MOBID	[47]	–	–
OPS-NVI	[48]	–	One item showed floor effect Nine items showed ceiling effect
	[49]	–	14 items showed floor effect One item showed ceiling effect
	[57]	–	–

†Theoretically, the FACS does not have a maximum score

The feasibility of using the included measurement instruments was evaluated based on the three main factors, which are the time required to complete the measurements using these instruments, the training provided before using the instruments and the tools required for undertaking the measurements. Findings about the feasibility are presented in Table 5.

**Table 5:** Feasibility of measurement instruments

Instrument name	Study	Training provided	Required tools	Completion time (Minutes)
*Oral health measurement instruments*				
BOHSE	[19]	Two 2-h training sessions	Tongue blade, hand-held light, gauze square and disposable gloves	Range = 5.0–20.0 Mean = 7.4
MPS	[43]	One to two hours training session	Headlight or hand-held light and two dental mirrors	Range = 2.0–4.0
THROAT	[21]	–	Hand-held light and gloves	NR
	[50]	Online training package, as well as hands-on training	–	Range = 1.0–5.0 Mean = 2.1
ROAG	[51]	3-h training sessions	Hand-held light and dental mirrors	–
	[44]	A visual guide was studied by the participants	Hand-held light and dental mirrors	–
OHAT	[20]	3-h training programme	Gloves	Range = 1.0–30.0 Mean = 7.8
	[52]	3-h training programme	Gloves	Range = 0.4–6.2 Mean = 2.5
	[53]	The first examiner, who has an extensive experience in using the OHAT, trained the second examiner	An abeslang (tongue spatula) and natural light	–
	[37]	Half-hour training	Hand-held light and gloves	–
OHI	[54]	The participants were trained by the research team	–	–
OAS	[36]	The participants were trained by the research team	–	–
OHSTNP	[39]	OHSTNP was used without any training	Hand-held light, tongue blades and dental mirrors	Range = 1.9–3.1 Mean = 2.6
BOE	[55]	Assessment was done by a dentist without any training	–	–
*Orofacial pain measurement instruments*				
FACS	[56]	The participant is certified FACS coders	–	–
MOBID	[47]	–	–	–
OPS-NVI	[48]	Standard instructions of using the OPS-NVI	–	–
	[49]	Standard instructions of using the OPS-NVI	–	–
	[57]	–	–	Mean = 12

## Discussion

This systematic review identified nine oral health and three orofacial pain measurement instruments that have been developed for use on different populations of dependent adults. Only the construct validity, responsiveness and reliability proprieties were evaluated for these instruments.

While only a limited number of the included studies in this review have reported evaluating the content validity of their measurement instruments [19–21], the outcomes of their evaluations were not incorporated into this systematic review synthesis. This is mainly because these studies, indeed, evaluated only the face validity of their measurement instruments. In addition, these evaluations were often undertaken by experts who were part of the development teams, and therefore, can be presumed to have a biased view toward their instruments. Furthermore, neither the method used to evaluate the face validity in these studies, nor the outcomes of the evaluations were reported fully or explicitly. It is widely accepted that to undertake a sound evaluation of a measurement instrument’s content validity, a qualitative study utilising the cognitive interviews approach is needed to be conducted with independent group of experts regarding three main criteria (i.e. relevance, comprehensive and comprehensibility) concerning the measurement instrument [31].

Final conclusions about many of the identified measurement instruments regarding construct validity and responsiveness were not established due to the very low quality of evidence supporting their findings. The very low quality of evidence could be attributed to the small sample size in many of these studies. For example, only 32 subjects were recruited to assess the construct validity of the THROAT. It has been suggested that at the last 50 subjects are need to establish an acceptable confidence interval around the estimated validity parameter [32]. In addition, as the hypotheses in all the contributing studies were generic and were not developed according to existing theories and relevant data pertaining to oral health and orofacial pain in dependent adults, they may not correctly reflect the true magnitude and direction of correlation or change in scores. Moreover, these studies did not establish their hypotheses in advance prior to data calculation, which could have led to potential biases during their analysis. Only the OPS-NVI demonstrated evidence of sufficient construct validity in this systematic review.

The OHAT and the THROAT instruments have been shown to have sufficient reliability, while most other instruments have been demonstrated to have either insufficient or inconsistent reliability. There are two possible sources that can be responsible for the insufficient and inconsistent reliability.

First, the poor reliability in the performance of these instruments could be attributed to a possibly large variance in their measurements due to the measurement error. While the values of measurement error were not reported for any of these instruments, there are indications supporting this explanation. The large variance due to the measurement error could occur when the participants who have undertaken these measurements do not have the adequate knowledge and skills to produce consistent measurements (i.e. among themselves and with others) [33]. In fact, most of the measurements (i.e. obtained with the instruments with poor reliability) were undertaken by nursing staff (i.e. BOHSE and Revised Oral Assessment Guide (ROAG)) and health care workers (i.e. Mucosal-Plaque Score (MPS) and Oral Assessment Sheet (OAS)), who have been suggested to have insufficient knowledge and skills about dentistry or oral health [16]. This may suggest that in order to improve the reliability of these instruments, more training and calibration for the participants are needed before using them [34, 35]. The reliability of the OHAT and the OAS has been shown to significantly improve after training has been provided for participants with no dental background [36, 37]. In addition, the training that was provided for the nursing staff and health care workers in the studies that have shown to have sufficient reliability seems to be more comprehensive and better than most of the studies that did not show a similar reliability in performance.

An extensive training, however, may not be possible from a logistical perspective, especially if these measurement instruments to be implemented and used national wide [38]. Thus, developing an instrument that is simple and can meet the caregivers’ level of oral health knowledge and skills could be an alternative approach that may establish sufficient reliability without the need for prior training. This approach is supported by the findings from the OHSTNP reliability study, which has reported achieving an acceptable level of reliability without providing any training for their participants [39].

It must be noted that the variations in the measurements may be incorrectly attributed to measurement errors, while in fact, these variations represent an actual change in the construct that is being measured. This can occur when the time between the measurements are relatively too long, and thus leads to a high chance for the measured construct to change between the different measurements [32]. This might have occurred in some of the included studies in this systematic review. As it is usually advisable to leave a two-week gap when assessing the intra-rater reliability, the constructs of oral health and orofacial pain could significantly change during this time period and thereby compromise the reliability of the instruments [40–42]. In fact, this reason was suggested by Henriksen (1999) and Konradsen et al. (2014) to explain the insufficient reliability of the MPS and the ROAG, respectively [43, 44].

The second source for the poor reliability of some measurement instruments in this systematic review could be due to the high homogeneity (i.e. in regard to the status of oral health and orofacial pain) among the samples of dependent adults within the included studies [45]. As the reliability is a ratio of the variance due to true differences in comparison to the variance due to measurement error, a small variation due to measurement error can significantly compromise the result of reliability if the sample is highly homogeneous (i.e. variance due to true changes is relatively small). Homogeneous samples could occur in studies when there are biases in the selection of the participants [46]. For example, Yanagisawa et al. (2017) utilised a self-selecting sampling method, which may have resulted in including dependent adults who have the highest interest in oral health among their sampling frame [36], which may explain why the OAS has failed to demonstrate sufficient reliability. However, even when the samples are highly heterogeneous, the variance stemming from true difference could be relatively small if the instrument is not adequately sensitive when measuring the construct of interest [32]. For example, Kayser-Jones et al. (1995) purposely selected patients with severe cognitive impairment in order to increase the heterogeneity among their sample [19]. However, they still failed to demonstrate a sufficient level of reliability for the BOHSE, because the BOHSE might not be sensitive enough to discriminate between the participants with different levels of oral health status. This could be supported by the reported mean and standard deviation of the BOHSE scores, which have placed most participants on the healthy side of the BOHSE scale.

Although the reliability of the OHSTNP and the Mobilization–Observation–Behaviour–Intensity–Dementia for mouth care (MOBID) have been evaluated, final conclusions could not be drawn, because the level of evidence quality was very low for these two instruments. The very low level of evidence was attributed to the small sample size in both studies [39, 47]. In addition, both studies suffered from many methodological flaws that increased their risk of biases. For example, the reliability of the OHSTNP was evaluated using inappropriate statistical parameter [39]. In contrast, Toxopeus et al. (2016) collected the data from 12 observers, and then only used the data from the best three observers to evaluate the reliability, which may have resulted in overestimating the reliability of the MOBID [47].

Even though none of the included studies in this systematic review has been specifically undertaken to evaluate interpretability or feasibility of their measurement instruments, some of the reported data from the included studies could be used to indirectly assess these two properties. Interpretability of the included measurement instruments in this review was evaluated based on the distribution of their scores. Most of the standard deviations of the included measurement instruments’ scores were relatively small, which may indicate that a small change in a score could reflect a substantial change in the construct that is being measured. However, because the characteristics of the samples in the included studies were usually not extensively described, it would be extremely difficult to distinguish if the small standard deviations reflect interpretability characteristics of the measurement instruments or only representing homogeneity of the samples in the included studies. The interpretability of the OPS-NVI was assessed by evaluating floor and ceiling effects. However, the two studies that assessed these effects were not consistent, which may be attributed to the differences between their samples [48, 49].

Data about the required training, required tools and the required time to undertake a measurement were used in this review to assess the feasibility of the included measurement instruments. While several instruments in this systematic review required minimal or no training at all (e.g. OHSTNP), others required an extensive training priori to their use (e.g. BOHES, ROAG and OHAT), which might have significantly compromised their feasibility [38]. In addition, several studies suggested that dental mirrors are needed when using their measurement instruments (e.g. MPS, ROAG, OHSTNP), which may not be available in many settings such as care homes, and thus reduced these instruments feasibility [14]. Lastly, some of the included instruments in this systematic review required an extended time to be completed (e.g. the OPS-NVI), and thus they may not be feasible to be used routinely for oral care planning [14].

While many steps were undertaken to ensure the method’s strength in this systematic review, there are still limitations that may have impact upon its internal and external validity. First, since all the included measurement instruments in this review were developed and tested on dependent elderly who are living in hospitals or care homes, it might not be appropriate to generalise the findings and conclusions of this systematic review beyond the tested populations. Indeed, this especially holds true for the conclusions about the orofacial pain measurement instruments because all these instruments have been specifically developed for patients with dementia. The second potential source for biases is the widespread case of methodological flaws among the included studies. This has limited the possibility to appraise the performances of many of the included measurement instruments, as the final evaluations of these instruments were unknown due to the quality of data presented in this respect. Moreover, due to the reasons related to feasibility, the search in this systematic review was restricted to the English language, which might lead to the introduction of language-related biases. Including relevant studies that were published in other languages may allow for the identification of more oral health and orofacial pain measurement instruments. In addition, including non-English studies may also improve the quality of the evidence about measurement properties for the included measurement instruments, and thus allow establishing more robust conclusions. Lastly, because there is no registration of studies of measurement properties, interpretability and feasibility as there is for randomised clinical trials [32], it was not possible to assess the impact of publication bias on the results of this systematic review.

## Conclusions

This systematic review revealed that there are nine oral health and three orofacial pain measurement instruments for dependent adults. However, none of these measurement instruments were shown to have been adequately and comprehensively tested to establish strong evidence in relation to their measurement properties, feasibility and interpretability. Nevertheless, some of the included measurement instruments in this review demonstrated sufficient performances in reliability (i.e. OHAT and THROAT) and construct validity (i.e. OPS-NVI). Thus, these instruments have the potential for future use once other measurement properties, interpretability and feasibility have been sufficiently tested and evaluated.

## Supplementary Information

**Additional** **file 1.** MEDLINE search strategy to identify relevant studies in the systematic review.

**Additional** **file 2.** Criteria for good measurement properties used in the systematic review.

**Additional file 3.** Main characteristics of the identified measurement instruments.

## Data Availability

Data analysed during this study was included in this article and its supplementary information files.

## Abbreviations

BA: Bana Abdulmohsen
BOE: Bedside Oral Examination
BOHSE: Brief Oral Health Status Examination
CENTRAL: Cochrane Central Register of Controlled Trials
COSMIN: Consensus-based Standards for the Selection of Health Measurement Instruments
FACS: Facial Actions Coding System
FB: Fahad BaHammam
GM: Giles McCracken
GRADE: Grading of Recommendation Assessment, Development and Evaluation
HTA: Health Technology Assessment
MeSH: Medical Subject Headings
MOBID: Mobilization–Observation–Behaviour–Intensity–Dementia for mouth care
MPS: Mucosal-Plaque Score
OAS: Oral Assessment Sheet
OATD: Open Access Theses and Dissertations
OHAT: Oral Health Assessment Tool
OHI: Oral Health Index
OHSTNP: Oral Health Screening Tool for Nursing Personnel
OPS-NVI: Orofacial Pain Scale for Non-Verbal Individuals
PICOS: Participants, Interventions, Comparators, Outcomes and Studies
PRISMA: Preferred Reporting Items for Systematic Reviews and Meta-Analyses
PROSPERO: The International Prospective Register of Systematic Reviews
ROAG: Revised Oral Assessment Guide
THROAT: The Holistic and Reliable Oral Assessment Tool
