# Supplementary material for: Measurement properties, interpretability and feasibility of instruments measuring oral health and orofacial pain in dependent adults: a systematic review
Source: BMC Oral Health. 2022 May 25;22:208. doi: 10.1186/s12903-022-02235-w (PMC9131695; doi:10.1186/s12903-022-02235-w)
Supplement: Supplementary file 1 — Additional file 1. MEDLINE search strategy to identify relevant studies in the systematic review. [file 12903_2022_2235_MOESM1_ESM.docx]

**MEDLINE search strategy to identify relevant studies in the systematic review**

| 1 | Oral Health/ or Stomatognathic Diseases/ or Jaw Diseases/ or exp jaw, edentulous/ or mandibular diseases/ or maxillary diseases/ or Mouth Diseases/ or Burning Mouth Syndrome/ or Candidiasis, Oral/ or Lip Diseases/ or Oral Ulcer/ or Periodontal Diseases/ or Salivary Gland Diseases/ or Stomatitis/ or Tongue Diseases/ or exp Temporomandibular Joint Disorders/ or Tooth Diseases/ or exp dental deposits/ or dentin sensitivity/ or focal infection, dental/ or exp mouth, edentulous/ or exp tooth demineralization/ or tooth loss/ or toothache/ or exp Dental Health Services/ or Dentistry/ or Dental Research/ or Evidence-Based Dentistry/ or Geriatric Dentistry/ or Preventive Dentistry/ or exp Oral Hygiene/ or Public Health Dentistry/ or exp Dental Health Surveys/ or Community Dentistry/ or (Oral* adj1 health*).mp. or (Oral* adj1 hygiene*).mp. or (Oral* adj1 disease*).mp. or (dent* adj1 disease*).mp. or (oral* adj2 care*).mp. or (mouth* adj2 care*).mp. or (t??th* adj2 care*).mp. or (T??th adj1 brush*).mp. or exp Facial Pain/ or (Orofacial adj2 pain).mp. or (Dent* adj2 pain).mp or (T??th adj2 pain).mp. or (oral* adj2 pain).mp or (mouth* adj2 pain).mp. or (facial* adj2 pain).mp. or TMD.mp. or (Temporomandibular adj1 disorder).mp. or T??thache.mp. or (Dental* adj1 care*).mp. or exp Jaw Neoplasms/ or Mucositis/ or Gingival Diseases/ or Periodontitis/ or Xerostomia/ or dental pulp diseases/ or Diagnosis, Oral/ or Photography, Dental/ or Cheilitis/ or Lip Neoplasms/ or Mouth Neoplasms/ or Gingival Neoplasms/ or Tongue Neoplasms/ or Stomatitis, Denture/ or tooth wear/ or tooth injuries/ or Tooth Fractures/ or (oral* adj1 ass*).mp. or (dent* adj1 ass*).mp. |
| --- | --- |
| 2 | Frail Elderly/ or Multiple Chronic Conditions/ or Catastrophic Illness/ or exp basal ganglia diseases/ or exp brain damage, chronic/ or exp brain death/ or exp brain diseases, metabolic/ or exp brain injuries/ or exp cerebellar diseases/ or exp cerebrovascular disorders/ or exp dementia/ or exp "diffuse cerebral sclerosis of schilder"/ or exp encephalitis/ or exp hypoxia, brain/ or exp leukoencephalopathies/ or exp neuroaxonal dystrophies/ or exp sepsis-associated encephalopathy/ or exp thalamic diseases/ or Disabled Persons/ or Amputees/ or Mentally Disabled Persons/ or Mentally Ill Persons/ or Motor Disorders/ or Intellectual Disability/ or Motor Skills Disorders/ or Developmental Disabilities/ or Neurocognitive Disorders/ or Consciousness Disorders/ or exp Coma/ or Persistent Vegetative State/ or Stupor/ or exp Cognition Disorders/ or Terminally Ill/ or Vulnerable Populations/ or Intensive Care Units/ or Burn Units/ or Coronary Care Units/ or Respiratory Care Units/ or exp Residential Facilities/ or Caregivers/ or Caregivers/ or Health Services for Persons with Disabilities/ or Critical Care Nursing/ or Geriatric Nursing/ or Neuroscience Nursing/ or Rehabilitation Nursing/ or Palliative Care Nursing/ or exp Critical Care/ or exp Institutionalization/ or Life Support Care/ or Long-Term Care/ or Subacute Care/ or exp Terminal Care/ or Palliative Care/ or Activities of Daily Living/ or Self care/ or Geriatric Assessment/ or (Dependent adj1 adult*).mp. or ICU.mp. or Disable*.mp. or ADL*.mp. or CAREGIVER*.mp. or Stroke*.mp. or Dementia*.mp. or Dementia*.mp. or Adult Day Care Centers/ or Inpatients/ |
| 3 | (instrumentation or methods).sh. or (Validation Studies or Comparative Study).pt. or exp Psychometrics/ or psychometr*.ti,ab. or (clinimetr* or clinometr*).tw. or exp "Outcome Assessment (Health Care)"/ or outcome measure*.tw. or exp Observer Variation/ or observer variation.ti,ab. or exp Health Status Indicators/ or exp "Reproducibility of Results"/ or reproducib*.ti,ab. or exp Discriminant Analysis/ or (reliab* or unreliab* or valid* or coefficient or homogeneity or homogeneous or "internal consistency").ti,ab. or (cronbach* and (alpha or alphas)).ti,ab. or (item and (correlation* or selection* or reduction*)).ti,ab. or (agreement or precision or imprecision or "precise values" or test-retest).ti,ab. or (test and retest).ti,ab. or (reliab* and (test or retest)).ti,ab. or (stability or interrater or inter-rater or intrarater or intra-rater or intertester or inter tester or intratester or intra-tester or interobserver or inter-observer or intraobserver or intra-observer or intertechnician or inter-technician or intratechnician or intra technician or interexaminer or inter-examiner or intraexaminer or intra-examiner or interassay or inter-assay or intraassay or intra-assay or interindividual or inter individual or intraindividual or intra-individual or interparticipant or inter-participant or intraparticipant or intra-participant or kappa or kappa's or kappas or repeatab*).ti,ab. or ((replicab* or repeated) and (measure or measures or findings or result or results or test or tests)).ti,ab. or (generaliza* or generalisa* or concordance).ti,ab. or (intraclass and correlation*).ti,ab. or (discriminative or "known group" or factor analysis or factor analyses or dimension* or subscale*).ti,ab. or (multitrait and scaling and (analysis or analyses)).ti,ab or (item discriminant or interscale correlation* or error or errors or "individual variability").ti,ab. or (variability and (analysis or values)).ti,ab. or (uncertainty and (measurement or measuring)).ti,ab. or ("standard error of measurement" or sensitiv* or responsive*).ti,ab. or ((minimal or minimally or clinical or clinically) and (important or significant or detectable) and (change or difference)).ti,ab. or (small* and (real or detectable) and (change or difference)).ti,ab. or (meaningful change or "ceiling effect" or "floor effect" or "Item response model" or IRT or Rasch or "Differential item functioning" or DIF or "computer adaptive testing" or "item bank" or "cross-cultural equivalence").ti,ab. |
| 4 | 1 and 2 and 3 |
| 5 | (child* not adult*).mp. |
| 6 | limit 5 to "all child (0 to 18 years)" |
| 7 | 4 not 6 |
| 8 | limit 7 to English language |
