# Supplementary material for: Measurement properties, interpretability and feasibility of instruments measuring oral health and orofacial pain in dependent adults: a systematic review
Source: BMC Oral Health. 2022 May 25;22:208. doi: 10.1186/s12903-022-02235-w (PMC9131695; doi:10.1186/s12903-022-02235-w)
Supplement: Supplementary file 2 — Additional file 2. Criteria for good measurement properties used in the systematic review. [file 12903_2022_2235_MOESM2_ESM.docx]

| **Criteria for good measurement properties used in the systematic review** | | |
| --- | --- | --- |
| **Measurement property** | **Rating** | **Criteria** |
| **Content validity** | **+** | The relevance rating is +, the comprehensive rating is + and the comprehensibility rating is + |
|  | **?** | Inconsistent ratings |
|  | **–** | The relevance rating is **–**, the comprehensive rating is **–** and the comprehensibility rating is **–** |
| **Internal consistency** | **+** | At least low evidence for sufficient structural validity AND Cronbach's alpha(s) ≥ 0.70 for each unidimensional scale or subscale |
|  | **?** | Criteria for at least low evidence for sufficient structural validity was not met |
|  | **–** | At least low evidence for sufficient structural validity AND Cronbach’s alpha(s) < 0.70 for each unidimensional scale or subscale |
| **Reliability** | **+** | ICC or Kappa ≥ 0.70 |
|  | **?** | ICC or Kappa not reported |
|  | **–** | ICC or Kappa < 0.70 |
| **Measurement error** | **+** | SDC or LoA < MIC |
|  | **?** | MIC not defined |
|  | **–** | SDC or LoA > MIC |
| **Construct validity** | **+** | The result is in accordance with the hypothesis OR AUC ≥ 0.70 |
|  | **?** | No hypothesis defined (by the research team or the review team) |
|  | **–** | The result is not in accordance with the hypothesis OR AUC < 0.70 |

| **Measurement property** | **Rating** | **Criteria** |
| --- | --- | --- |
| **Structural validity** | **+** | **CTT:** CFA: CFI or TLI >0.95 OR RMSEA <0.06 OR SRMR <0.082  **IRT/Rasch:** No violation of unidimensionality: CFI or TLI >0.95 OR RMSEA <0.06 OR SRMR <0.08  *AND* no violation of local independence: residual correlations, among the items after controlling for the dominant factor < 0.20 OR Q3s < 0.37  *AND* no violation of monotonicity: adequate looking graphs OR item scalability >0.30  *AND* adequate model fit: IRT: χ2 >0.01  Rasch: infit and outfit mean squares ≥ 0.5 and ≤ 1.5 OR Z-standardized values > –2 and < 2 |
|  | **?** | CTT: Not all information for ‘+’ reported  IRT/Rasch: Model fit not reported |
|  | **–** | Criteria for ‘+’ not met |
| **Cross-cultural**  **validity** | **+** | No important differences found between group factors (such as age, gender, language) in multiple group factor analysis OR no important DIF for group factors (McFadden's R^2^ < 0.02) |
|  | **?** | No multiple group factor analysis OR DIF analysis performed |
|  | **–** | Important differences between group factors OR DIF were  found |
| **Criterion validity** | **+** | Correlation with gold standard ≥ 0.70 OR AUC ≥ 0.70 |
|  | **?** | Not all information for ‘+’ reported |
|  | **–** | Correlation with gold standard < 0.70 OR AUC < 0.70 |
| **Responsiveness** | **+** | The result is in accordance with the hypothesis OR AUC ≥ 0.70 |
|  | **?** | No hypothesis defined (by the research team or the review team) |
|  | **–** | The result is not in accordance with the hypothesis OR AUC < 0.70 |

| - **Abbreviations used in the table:**   - ‘+’ = sufficient, ‘–’ = insufficient, ‘?’ = indeterminate   - AUC = Area Under the Curve, CFA = Confirmatory Factor Analysis, CFI = Comparative Fit Index, CTT = Classical Test Theory, DIF = Differential Item Functioning, ICC = Intraclass Correlation Coefficient, IRT = Item Response Theory, LoA = Limits of Agreement, MIC = Minimal Important Change, RMSEA: Root Mean Square Error of Approximation, SEM = Standard Error of Measurement, SDC = Smallest Detectable Change, SRMR: Standardized Root Mean Residuals, TLI = Tucker-Lewis index - **Generic hypotheses that were used to evaluate construct validity and responsiveness** (Prinsen *et al.*, 2018):   - Correlations with (changes in) instruments measuring similar constructs should be ≥0.50   - Correlations with (changes in) instruments measuring related, but dissimilar, constructs should be lower, i.e. 0.30–0.50   - Correlations with (changes in) instruments measuring unrelated constructs should be <0.30   - Meaningful changes between relevant (sub)groups (e.g. patients with expected high vs. low levels of the construct of interest) |
| --- |

These criteria were adapted from Terwee *et al.* (2007) and Terwee *et al.* (2018).
