# Supplementary material for: Measurement properties, interpretability and feasibility of instruments measuring oral health and orofacial pain in dependent adults: a systematic review
Source: BMC Oral Health. 2022 May 25;22:208. doi: 10.1186/s12903-022-02235-w (PMC9131695; doi:10.1186/s12903-022-02235-w)
Supplement: Supplementary file 3 — Additional file 3. Main characteristics of the identified measurement instruments. [file 12903_2022_2235_MOESM3_ESM.docx]

| **Main characteristics of the identified measurement instruments** | | | | | | | | |
| --- | --- | --- | --- | --- | --- | --- | --- | --- |
| **Main characteristics of the identified oral health measurement instruments.** | | | | | | | | |
| **Instrument name** | **Language** | **Number of items** | **Scale type** | **Instrument type** | **Scoring algorithm** | **Instrument aim** | **Users** | **Target population** |
| **BOHSE** | English | 10 | 3-point | Clinician-reported | Simple sum | Oral care planning | Nursing staff | Care home residents |
| **MPS** | English Norwegian | 2 | 4-point | Clinician-reported | Simple sum | Group assessment | Health care workers | Care home residents |
| **THROAT** | English | 9 | 4-point | Clinician-reported | Not applicable | Oral care planning | Nursing staff | Hospitalised patients |
| **ROAG** | English Danish Swedish | 8 | 3-point | Clinician-reported | Simple sum | Oral care planning | Nursing staff | Hospitalised patients |
| **OHAT** | English German  Turkish | 8 | 3-point | Clinician-reported | Simple sum | Oral care planning | Health care workers and Speech language therapists | Care home residents and hospitalised patients |
| **OHI** | English French | 8 | Dichotomous | Clinician-reported | Simple sum | Oral care planning | Nursing staff | Care home residents |
| **OAS** | English Japanese | 9 | 3-point | Clinician-reported | Simple sum | Oral care planning | Health care workers | Care home residents |
| **OHSTNP** | English Japanese | 12 | 3-point | Clinician-reported | Simple sum | Oral care planning | Nursing staff and health care workers | Care home residents |
| **BOE** | English  Danish | 8 | 3-point | Clinician-reported | Simple sum | Oral care planning | Nursing staff | Hospitalised patients |
| **Main characteristics of the identified orofacial pain measurement instruments.** | | | | | | | | |
| **FACS** | English | 6 | Not applicable | Clinician-reported | A FACS score consisted of adding the duration of eye closing to the outcome of multiplying other items’ intensity by their durations | Oral care planning | Dentists | Elderly with communication difficulties |
| **MOBID** | English | 3 | Dichotomous | Clinician-reported | Not applicable | Oral care planning | Dentists | Elderly with communication difficulties |
| **OPS-NVI** | English Dutch | 64 | Dichotomous | Clinician-reported | Simple sum | Oral care planning | Dentists | Elderly with communication difficulties |
